# In Vitro Mechanical and Biological Properties of 3D Printed Polymer Composite and β-Tricalcium Phosphate Scaffold on Human Dental Pulp Stem Cells

**DOI:** 10.3390/ma13143057

**Published:** 2020-07-08

**Authors:** Shuaishuai Cao, Jonghyeuk Han, Neha Sharma, Bilal Msallem, Wonwoo Jeong, Jeonghyun Son, Christoph Kunz, Hyun-Wook Kang, Florian M. Thieringer

**Affiliations:** 1 Department of Oral and Cranio-Maxillofacial Surgery, University Hospital Basel, Spitalstrasse 21, 4031 Basel, Switzerland; shuaishuai.cao@unibas.ch (S.C.); neha.sharma@usb.ch (N.S.); bilal.msallem@usb.ch (B.M.); christoph.kunz@usb.ch (C.K.); 2 Medical Additive Manufacturing Research Group, Department of Biomedical Engineering, University of Basel, Gewerbestrasse 16, 4123 Allschwil, Switzerland; 3 Biomedical Engineering, School of Life Sciences, Ulsan National Institute of Science and Technology (UNIST), 50, UNIST-gil, Ulsan 44919, Korea; g14619@unist.ac.kr (J.H.); wwjeong@unist.ac.kr (W.J.); mgm4336@unist.ac.kr (J.S.)

**Keywords:** 3D printing, dental biomaterials, polymer printing, ceramic printing, human dental pulp stem cell, in vitro research, bone regeneration

## Abstract

3D printed biomaterials have been extensively investigated and developed in the field of bone regeneration related to clinical issues. However, specific applications of 3D printed biomaterials in different dental areas have seldom been reported. In this study, we aimed to and successfully fabricated 3D poly (lactic-co-glycolic acid)/β-tricalcium phosphate (3D-PLGA/TCP) and 3D β-tricalcium phosphate (3D-TCP) scaffolds using two relatively distinct 3D printing (3DP) technologies. Conjunctively, we compared and investigated mechanical and biological responses on human dental pulp stem cells (hDPSCs). Physicochemical properties of the scaffolds, including pore structure, chemical elements, and compression modulus, were characterized. hDPSCs were cultured on scaffolds for subsequent investigations of biocompatibility and osteoconductivity. Our findings indicate that 3D printed PLGA/TCP and β-tricalcium phosphate (β-TCP) scaffolds possessed a highly interconnected and porous structure. 3D-TCP scaffolds exhibited better compressive strength than 3D-PLGA/TCP scaffolds, while the 3D-PLGA/TCP scaffolds revealed a flexible mechanical performance. The introduction of 3D structure and β-TCP components increased the adhesion and proliferation of hDPSCs and promoted osteogenic differentiation. In conclusion, 3D-PLGA/TCP and 3D-TCP scaffolds, with the incorporation of hDPSCs as a personalized restoration approach, has a prospective potential to repair minor and critical bone defects in oral and maxillofacial surgery, respectively.

## 1. Introduction

Oral and maxillofacial bone defects caused by trauma, tumors, and malformations are common clinical scenarios causing physiological and psychological afflictions to the patients. With complex bone defect architectures and unique functions, reconstructive possibilities in this field are still a challenge for the current states of available clinical treatments. Ultimately, the goal of oral and maxillofacial bone reconstruction is to imitate and reconstruct the original anatomical features to provide desirable long-term functional and esthetical results [1].

In the past decade, many techniques, such as forming, subtractive manufacturing, electrospinning, or combinations of these processes have been applied to fabricate artificial bone substitutes [2,3,4]. However, these methods have several limitations, for example, low controllability of internal pore design (i.e., pore size, pore-interconnectivity, and tortuosity, etc.) and limitations in creating specific three-dimensional (3D) shapes [1,5]. To overcome the limitations of these methods, 3D printing (3DP) has been applied. 3DP technology is contrary to traditional “subtractive manufacturing” technology whereby there is direct processing of materials according to the principle of “additive manufacturing” and layer-by-layer superposition [6]. This manufacturing process allows the precise control of the porous geometry and internal architecture of the 3D printed object. Importantly, 3DP techniques can incorporate imaging techniques, such as computed tomography (CT) and magnetic resonance imaging (MRI), to generate patient-specific 3D tissue models [7]. Such approaches can be used to provide a blueprint for fabricating customized bone scaffolds with accurate anatomical shapes used in oral and maxillofacial bone defects.

Most clinically used patient-specific implants for oral and maxillofacial reconstruction are composed of titanium, poly-ether-ether-ketone (PEEK), and other non-absorbable materials [6,8,9]. However, to achieve optimal scaffolding to facilitate regeneration of hard tissues, various scaffold properties, such as controlled biodegradability, architecture, mechanical property, biocompatibility, and osteoconductivity, should be considered. One type of commonly used polymer in oral and maxillofacial surgeries is poly lactic-co-glycolic acid (PLGA), which has been influential in tissue engineering and has been FDA-approved since 1986 [10]. PLGA-based applications include drug delivery systems, biodegradable sutures, and scaffolds for bone tissue regeneration [11]. However, the mechanical properties of PLGA lack the desired and required stability for optimal bone tissue engineering. Nevertheless, because of the dual organic and inorganic nature of bone, and thanks to its versatility, PLGA can be mixed with other materials to create a compound with increased stability and additional reinforcement, such as when combined with tricalcium phosphate (TCP) [12,13]. TCP is composed of calcium-phosphate, its chemical formula is Ca_3_(PO_4_)_2_, and TCP naturally occurs inside the human body during the mineral phase for bone tissue [14]. Chen et al. [15] found that rhBMP-2 loaded 3D poly (lactic-co-glycolic acid)/β-tricalcium phosphate (3D-PLGA/TCP) scaffolds fabricated by low-temperature rapid prototyping technology repaired ulnar defects in rabbits. Abarrategi et al. [16] reported that the 3D β-tricalcium phosphate (3D-TCP) scaffold produced by 3DP performed a fully controllable and customizable structure and had good biocompatibility. It is evident that the existence of a balance between scaffold degradation and new bone formation is vital for better performance of scaffolds used in bone tissue engineering [17]. These are the main reasons why controlled degradation and better mechanical properties are needed to facilitate the development of usable advanced bone scaffolds, in parallel with innovations in bioresorbable materials.

Another essential aspect of a 3D printed scaffold is the seeding and co-culturing of the scaffolds with stem cells before implantation. To date, many studies have demonstrated that bone marrow mesenchymal stem cells (BMSCs) possess multilineage differentiation potential and can be widely used as seed cells for bone tissue engineering [18,19]. However, BMSCs are difficult to harvest, and its phenotypic behaviors are disabled during the culturing and expanding phases [20]. To counter these issues, in recent years, researchers have turned their attention to the use of human dental pulp stem cells (hDPSCs) instead. hDPSCs can be obtained from extracted wisdom teeth or teeth extracted due to orthodontic reasons, without additional injury. Some researchers have already isolated hDPSCs from adult human dental pulp and found that, like osteoblasts, hDPSCs expressed bone biomarkers such as type I collagen, alkaline phosphatase, and osteocalcin [18,21]. Furthermore, findings indicated that hDPSCs efficiently induced improvement in periodontal bone regeneration when implanted with conventionally made β-tricalcium phosphate (β-TCP) into swine [22]. Thus far, a lack of reporting of mechanical and biological comparison, especially with hDPSCs on different manufactured 3D-PLGA/TCP, 3D-TCP scaffolds, remains to be expanded.

**Figure 1:**
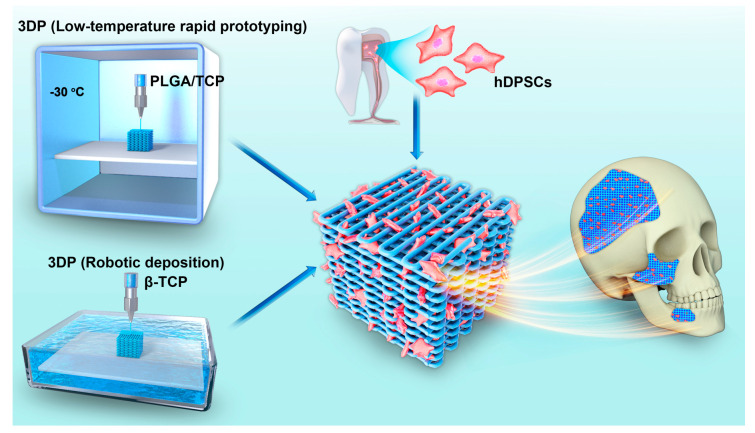
Overview of the application of two types of 3DP technologies used to fabricate scaffold incorporated with hDPSCs for oral and maxillofacial bone reconstruction.

In our current study, we utilized a preliminary approach to explore the feasibility of different 3D printed bioresorbable scaffolds to facilitate the reconstruction of patient-specific defects in oral and maxillofacial surgery. Thus, we sought to fabricate PLGA/TCP and β-TCP samples with the same morphologies, structures, and pore sizes (Figure 1). Besides, we also examined and compared the mechanical properties and biological behaviors of hDPSCs in these scaffolds in comparison to conventionally manufactured β-TCP scaffolds. We expect that our study could increase the understanding of the specific dental applications of 3D printed polymer composite and β-tricalcium phosphate scaffold in the field of oral and maxillofacial bone regeneration.

## 2. Materials and Methods

### 2.1. Scaffold Preparation

3D-PLGA/TCP cube (8 × 8 × 8 mm^3^) and cubic disk samples (8 × 8 × 2 mm^3^) were fabricated with a low-temperature rapid prototyping 3D printer (Tissue Form II, Tsinghua University, Beijing, China) controlled by the printer’s proprietary software program (Cark, Tsinghua University, Beijing, China). PLGA/TCP ink was prepared as described in a previous study [15]. Briefly, PLGA and β-TCP powder with a weight-based ratio of 75:25 was dissolved in 1,4-dioxane with magnetic stirring at 37 °C for 12 h. Then, the homogenized paste was added to the print head for subsequent printing. Table 1 lists the printing parameters we used for PLGA/TCP. Post-printing, the samples were dried in a vacuum freeze-dryer (LyoAlfa, Telstar, Terrassa, Spain) for solvent volatilization.

3D-TCP cube (8 × 8 × 8 mm^3^) and cubic disk samples (8 × 8 × 2 mm^3^) were fabricated with a robotic deposition 3D printer (3D Inks, Stillwater, MN, USA) controlled by Robocad 3.0 (3D Inks, Stillwater, MN, USA). β-TCP ink was prepared as described in a previous study [16]. Briefly, the β-TCP powder was mixed with deionized water, Darvan^®^ C dispersant (Vanderbilt Minerals, Darvan C-N, CT, USA), hydroxypropyl methylcellulose, and flocculant in a concentrator overnight. After that, the β-TCP ink was added to a metal print head for printing. Table 2 lists the printing parameters we used for β-TCP. Finally, the samples were dried at room temperature for 24 h and were then sintered at 400 °C and 1200 °C respectively for 1 h to evaporate organic constituents.

A commercialized porous β-TCP (Bio-lu Biomaterials, Shanghai, China) was purchased and then cut into a cube (8 × 8 × 8 mm^3^) and a cubic disk (8 × 8 × 2 mm^3^) to serve as samples for the control group. These were conventionally fabricated by forming technology with a fully interoperable globular pore structure, an open porosity of 70 (±10)%, and a sphericity aperture of 400 (±50) μm. 3D β-TCP (3D-TCP) and commercialized β-TCP (C-TCP) were sterilized via autoclaving at 134 °C for 5 min, whereas 3D-PLGA/TCP samples were sterilized by gamma radiation of cobalt 60 under 30 kGy, after which samples were stored at room temperature. 

### 2.2. Characterization of Scaffolds

The surface morphology and energy dispersive spectra (EDS) of the materials were examined with a scanning electron microscope (SEM; S-4800, Hitachi High-Technologies Co., Tokyo, Japan). A 20 nm thick Au-Pd film layer was uniformly sprayed on scaffolds surface prior to SEM analyses. Micro-CT (SkyScan 1172, Bruker, Belgium) was employed to qualitatively evaluate pore size and porosity with a scanning resolution of 15 μm (80 kV and 100 μA radiation source with a 0.5 mm aluminum filter). Volumetric reconstruction and porosity analyses were conducted separately with CTan 1.13 (NRecon 1.1, Bruker, Belgium).

The compressive mechanical properties of each sample were measured by a uniaxial compressive testing system (Electromechanical 3382, Instron, Norwood, MA, USA). C-TCP, 3D-PLGA/TCP, and 3D-TCP (N = 8) cubic samples (8 mm × 8 mm × 8 mm^3^) were prepared on the holder and crosshead speed was set at 1 mm/min. The maximum applied load was recorded and compressive strength (MPa) was subsequently calculated by dividing the maximum applied load by the initial cross-sectional area of the samples.

### 2.3. Biological Responses of hDPSCs

#### 2.3.1. Cell Culture and Seeding

The hDPSCs (Lonza, Walkersville, MD, USA) were cultured and expanded with Alpha-minimum eagle’s medium (α-MEM) supplemented with 10% fetal bovine serum (FBS; Gibco, Thermo Fisher Scientific, Waltham, MA, USA) and were supplemented with 1% v/v penicillin-streptomycin (P/S) in physiological conditions held constant (37 °C air temperature, 5% CO_2_ atmosphere). The mediums were exchanged every 3 days. Subculturing was conducted when samples reached 70%–80% confluency through dissociation induced with TrypLE™ Select (1×) (Gibco, Thermo Fisher Scientific, Waltham, MA, USA). Adherent cells on flask-bottoms were separated by 1.5 mL trypsin-EDTA (0.05% trypsin/0.02% EDTA, Life Technologies Co., Waltham, MA, USA) for 5 min at 37 °C in an incubator.

hDPSCs were prepared into cell suspensions and we adjusted cell concentrations to 1 × 10^6^ cells /mL. C-TCP, 3D-PLGA/TCP, and 3D-TCP (N = 8) samples were placed in a 24-well plate, and 15 μL of hDPSCs suspension was seeded onto each cubic disk for pre-culturing for 2 h. After cells were dropped to the scaffold, culture mediums were refilled.

#### 2.3.2. Live/Dead Staining

After culturing for three days, live/dead staining (L3224, Thermo Fisher Scientific, Waltham, MA, USA) was performed to facilitate measurements of cell viability for each group (C-TCP, 3D-PLGA/TCP and 3D-TCP, N = 4). Scaffolds were stained with assay solution (0.2% v/v calcein AM, and 0.05% v/v ethidium homodimer-1 in phosphate-buffered saline (PBS) at room temperature for 45 min and were then imaged using fluorescence microscopy (Leica DM2500, Leica Microsystems AG, Wetzlar, Germany). The live and dead cells were manually counted in live and dead staining images with Leica software (LAS X, Leica Microsystems AG, Wetzlar, Germany), and the cell viability was reported as a percentage by dividing the number of live cells by the total cell count.

#### 2.3.3. Cell Metabolic Activity

Cell viability reagent (AlamarBlue™, Thermo Fisher Scientific, Waltham, MA, USA) was employed to facilitate cell metabolic activity. After culturing for 1, 3, 5, and 7 days, three groups of scaffolds (N = 8 each group) were incubated in 10% v/v Alamar Blue dye diluted by culture medium at a constant temperature of 37 °C and with a constant 5% CO_2_ atmosphere for 3 h following all manufacturer protocols. After sampling assay solutions in 100-μL sized aliquots, their fluorescence intensities (excitation: 544 nm/emission: 599 nm) were measured spectrophotometrically with a microplate reader (Synergy NEO2 Hybrid Multi-Mode Reader, Bio-Tek, Winooski, VT, USA). Then, the measured data were normalized relative to data collected on day 1. Finally, the culture mediums were renewed at the end of each measurement.

#### 2.3.4. Osteogenic Differentiation

Alkaline phosphatase (ALP) activity was used to evaluate effects in different groups (C-TCP, 3D-PLGA/TCP and 3D-TCP, N = 8) on the differentiation of hDPSCs. After seeding and culturing for 24 h, culture mediums were replaced by osteogenic mediums (alpha-minimum eagle’s medium supplemented with 10%, fetal bovine serum, 10 mM b-glyceraldehyde-3-phosphate, 50 mg/mL L-ascorbic acid, and 10 nM dexamethasone) as previously described [22]. The mediums were changed every 3 days, and ALP activity was calculated both on day 3, 7 and 14 with an ALP assay kit (BioVision, Milpitas, CA, USA). For quantitative analysis, scaffolds were washed using PBS and then lysed in 0.1% Triton X-100 for 40 min. Lysates were then centrifuged at 2500 rpm for 10 min at 4 °C. Supernatant was collected in a 96-well plate, and an equal volume of p-nitrophenylphosphate (pNPP) substrate was added, the samples incubated at 37 °C for 1 h to allow cleavage of the chromogenic substrate following manufacturer’s protocol. The protein concentration of each sample was determined by the bicinchonic acid (BCA) protein assay kit (Best Bio, Shanghai, China) at optical density (OD) 540 nm and recorded according to the standard curve in order to normalize ALP activity. OD values of ALP activity were recorded at a wavelength of 405 nm. Finally, ALP activity was reported as ALP (Units/mg) = ($\frac{\mathrm{measure}\;\mathrm{OD}-\mathrm{blank}\;\mathrm{OD}}{\mathrm{standard}\;\mathrm{OD}-\mathrm{blank}\;\mathrm{OD}}\times \mathrm{standard}\;\mathrm{concentration}\;(0.1\;\mathrm{mg}/\mathrm{mL}))/P$, P (protein concentration).

### 2.4. Statistical Analysis

All data were presented as mean ± standard deviation (mean ± SD). Statistical analyses were performed using GraphPad Prism 7 (GraphPad Software, La Jolla, CA, USA). Statistical differences between two groups were assessed by using paired two-sided t tests. Statistical differences among multiple groups were evaluated by one-way analysis of variance (ANOVA) followed by Tukey’s post-hoc tests. The statistical significance level was defined at *p* < 0.05.

## 3. Results

### 3.1. Characterization of Scaffolds

#### 3.1.1. Structure and Surface

**Figure 2:**
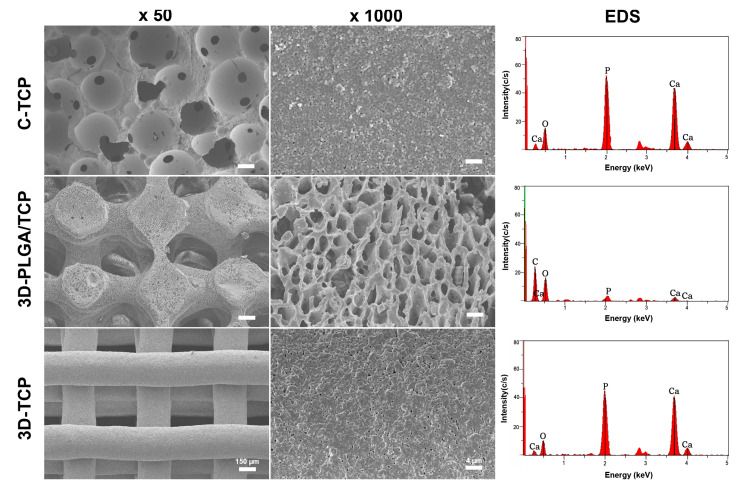
Characterizations of C-TCP, 3D-PLGA/TCP, and 3D-TCP scaffolds. SEM images showed the 3D interconnected structure and scaffold surface, and EDS-based assessments indicated the presence of β-TCP in these three scaffolds.

The structure and surface of C-TCP, 3D-PLGA/TCP, and 3D-TCP scaffolds were evaluated using SEM (Figure 2). Results indicated that all three groups of scaffolds possessed highly interconnected porous macro-structure and microporous structures on the surface. Compared with the C-TCP group, increasingly interconnected and homogeneous 3D pore structures were observed in the 3D-PLGA/TCP and 3D-TCP groups. Moreover, the 3D printed organic/inorganic PLGA/TCP compounds exhibited larger opening levels of connected micro-porosity on surfaces, compared with respective observations of rough surfaces for C-TCP and 3D-TCP groups. EDS-based results indicated that C-TCP and 3D-TCP scaffolds had similarly scaled presences of calcium (Ca) and phosphorus (P) peaks, whereas Ca and P peaks were decreased with corresponding decreased β-TCP proportions in the 3D-PLGA/TCP group.

Table 3 shows pore sizes and porosities of different groups measured by micro-CT. The pore size of conventionally manufactured β-TCP scaffold was measured as 375 (±30.3) μm, which was larger than the pore size of 3D printed PLGA/TCP and β-TCP scaffolds each respectively measured as 362 (±16.5) and 345 (±9.1) μm. These three different scaffolds exhibited similarly to more than 60% porosity. The 3D-PLGA/TCP scaffolds possessed lower porosity = 65.6 (±5.0)%, whereas the porosity of the C-TCP and 3D-TCP scaffolds were 67.4 (±2.7)% and for 3D-TCP were 72.5 (±2.5)%. These observations demonstrated that despite their differences in material composition, these three different scaffolds presented very similar pore sizes and porosities.

#### 3.1.2. Mechanical Properties

**Figure 3:**
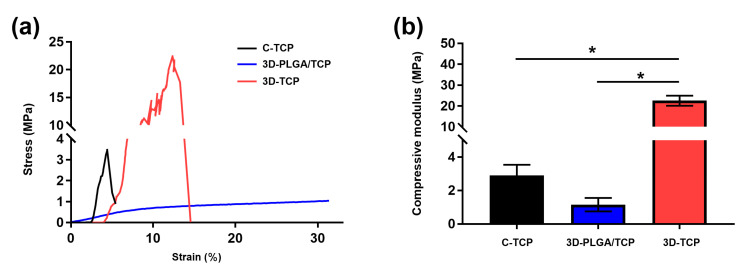
Mechanical properties of C-TCP, 3D-PLGA/TCP, and 3D-TCP scaffolds. (**a**) Compressive strain–stress curves indicated that 3D-PLGA/TCP possessed flexible mechanical properties. (**b**) Effects of 3DP and β-TCP amounts on the compressive modulus (* *p* < 0.050).

Representative compressive strain–stress curves and modulus value for different groups of scaffolds are shown in Figure 3. Results indicated that the C-TCP and 3D-TCP scaffolds exhibited higher compressive strength than the 3D-PLGA/TCP scaffold, whereas the 3D-TCP scaffold presented the best compressive modulus among the tested groups. Moreover, the compressive modulus of the 3D-TCP scaffold increased significantly compared to the C-TCP scaffold, likely because of the 3D layer-by-layer structure and even being composed of the same materials. Primary consideration should be given to the occurrences of fractures, which were not observed in the 3D-PLGA/TCP group when they had experienced up to 80% levels of strain. These findings demonstrated that 3D-PLGA/TCP composite scaffold possessed excellent flexibility. The compressive modulus of the 3D-PLGA/TCP scaffold was calculated at a 20% strain, which was a significant decrease compared to the measures respective to the 3D-TCP scaffold.

### 3.2. Biological Responses of hDPSCs

#### 3.2.1. Live/Dead Staining

**Figure 4:**
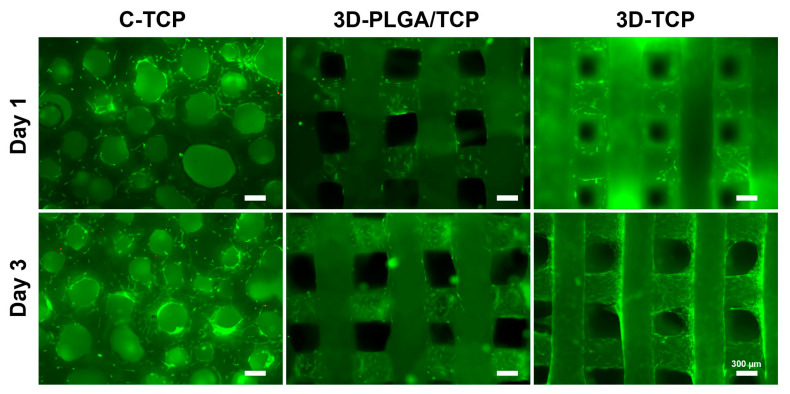
The effect of C-TCP, 3D-PLGA/TCP, and 3D-TCP scaffolds on hDPSCs viability. The live/dead assay results indicated that hDPSCs possessed excellent cell viability on the surfaces of all tested scaffolds (green: live cells; red: dead cells).

The adhesion morphology and viability of hDPSCs on different scaffolds were evaluated using fluorescent live/dead assays (Figure 4). Live hDPSCs (green-stained) attached on the surfaces of scaffolds, while only a few dead cells (red-stained) were observed on day 1 and 3. This suggested that the materials and designed structures possessed excellent cytocompatibility. At day 3, we can see that the hDPSCs adhered and proliferated deeper in 3D-PLGA/TCP and 3D-TCP scaffold, but the C-TCP material did not. This indicated that the well-connected and internal structure of the 3D printed material enhanced hDPSCs migration inside.

**Figure 5:**
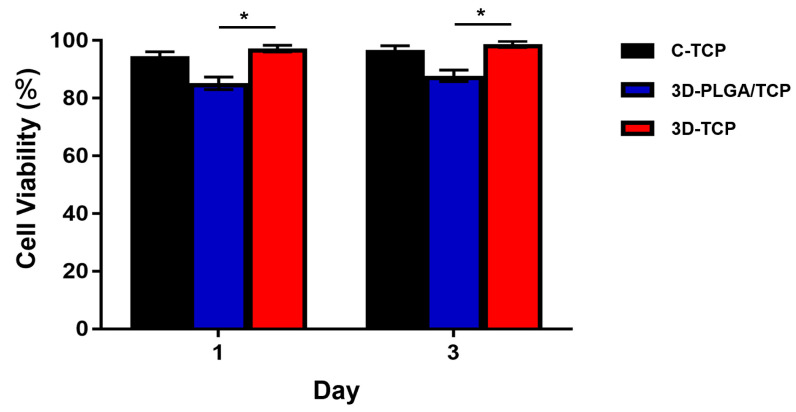
hDPSCs viability of C-TCP, 3D-PLGA/TCP, and 3D-TCP scaffolds. C-TCP and 3D-TCP scaffolds showed good cell viability over time, whereas 3D-PLGA/TCP scaffold had significantly lower cell viability than the other two groups (* *p* < 0.050).

The viability of hDPSCs is shown in Figure 5. In our result, C-TCP and 3D-TCP group showed a consistent trend of excellent cell viability over time, reaching more than 90% after 3 days in culture. Although hDPSCs viability of 3D-PLGA/TCP was significantly lower compared with 3D-TCP group, it is still higher than 80%.

#### 3.2.2. Cell Metabolic Activity

**Figure 6:**
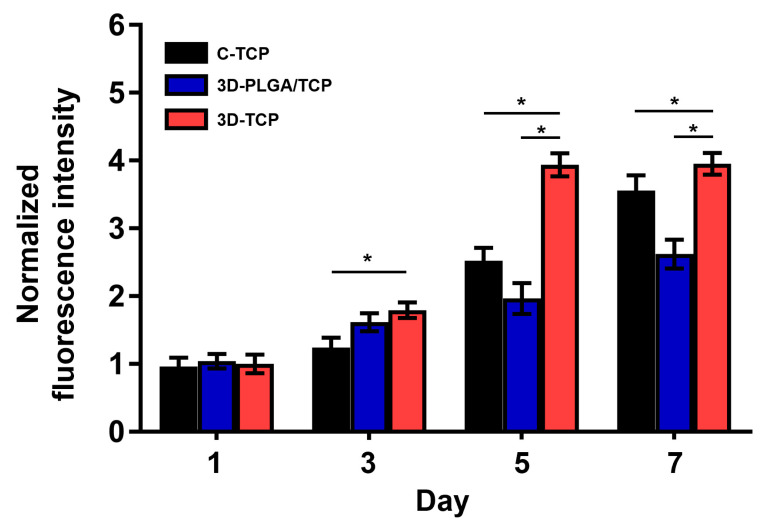
hDPSCs proliferation on C-TCP, 3D-PLGA/TCP, and 3D-TCP scaffolds. AlamarBlue assays indicated that all scaffolds significantly promoted the proliferation of hDPSCs (* *p* < 0.050).

The proliferation of hDPSCs on each group of scaffolds after 1, 3, 5, and 7 days of co-culturing are reported in Figure 6. In general, proliferation rates of hDPSCs in all groups increased over time. This result confirmed that C-TCP, 3D-PLGA/TCP, and 3D-TCP are quite feasible for use as carriers of hDPSCs. On day 1, there were no significant differences in these three groups. However, on day 3, the proliferation of 3D-PLGA/TCP and 3D-TCP groups were higher than of the C-TCP group. However, results also indicated that cell metabolic activity in the 3D-PLGA/TCP group was significantly lower than in the 3D-TCPgroup on days 3, 5, and 7. Additionally, a similar phenomenon was observed that the hDPSCs proliferation in the 3D-TCP group was higher after 3 days of co-culturing compared to C-TCP group.

#### 3.2.3. Osteogenic Differentiation

**Figure 7:**
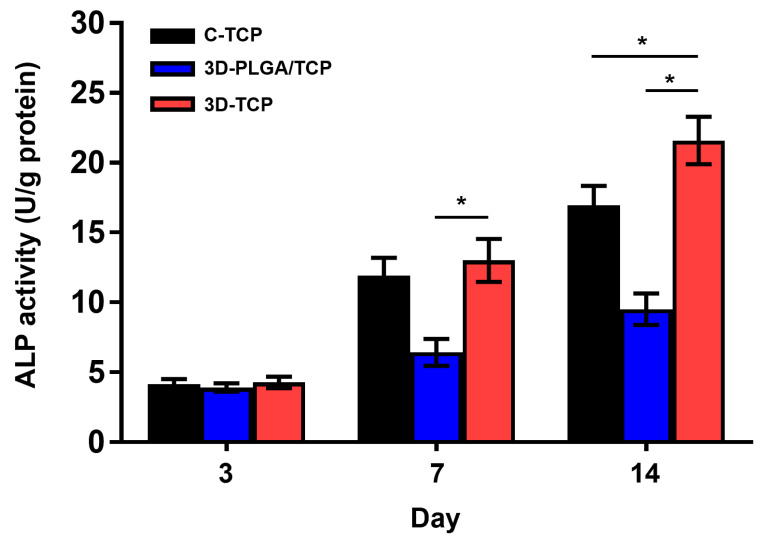
The effect of C-TCP, 3D-PLGA/TCP, and 3D-TCP scaffolds on ALP activity. The 3D interconnected structure and β-TCP amount enhanced the ALP activity on day 3, 7, and 14 (* *p* < 0.050).

BCA protein concentration (Figure S1) of each samples and quantitative ALP activity (Figure 7) indicated that there were no significant differences among all three groups and respective scaffolds on day 3. Furthermore, higher ALP activity values were revealed on the 3D-TCP scaffold compared to the 3D-PLGA/TCP and C-TCP scaffolds. However, the ALP activity of the 3D-PLGA/TCP group was significantly lower than in 3D-TCP group on day 7, and 14. Additionally, compared to the C-TCP scaffold, the 3D-TCP scaffold enhanced the expression of ALP activity. These results indicated that the osteogenic differentiation of hDPSCs favored β-TCP and 3D porous structure content.

## 4. Discussion

As a crucial issue in bone defect reconstruction, the mechanical and biological properties of PLGA/TCP and TCP scaffolds fabricated with two different printing technologies were assessed and compared in terms of specific oral and maxillofacial application. Once the chemical, physical, mechanical, and biological properties of the 3D printed biomaterials are well understood, then these biomaterials can be tailored to provide specific clinical applications. Comparison of differentially composed 3DP biomaterials with conventionally made biomaterials to facilitate examinations of mechanical property and biological responses on hDPSCs remains poorly investigated. In this study, we compared the mechanical properties of two 3D printed materials with a conventionally formed scaffold and assessed their biocompatibility and osteogenic differentiation on hDPSCs. The fact that rapid low-temperature prototyping and robocasting are extrusion-based and the widespread use of the layer-by-layer manufacturing process in 3DP helped to meet the needs of clinical applications [15,16]. Notably, in this regard, it can be seen in Figure 2 and Table 3 that the SEM and Micro-CT results indicated that two kinds of scaffolds with the same structures could be fabricated by each of the two 3DP technologies employed in this study. Besides, thanks to the advancement of 3DP technology, not only can the desired appearance be achieved, but also the internally controlled pore size and porosity can be achieved [3]. Our scaffold presented a macropore size of 300–400 μm, indicating that they were suitable for capillary formation, mineral deposition, and extracellular matrix secretion [13,15,16,23]. The in vitro results implied that 3D uniform architectures were an important contributing factor in achieving the desired levels of cell colonization, proliferation, and osteoconductivity. The highly porous and consistent structure also allowed for the 3D scaffold to be an appropriate carrier to facilitate the slow-release of drugs. This is an important consideration for future related research endeavors pursuant to our efforts.

Regarding targeting-based approaches for dental hard tissue regeneration, the mechanical behavior of the materials after 3DP has not been paid much attention. Weak mechanical properties of the scaffold are generally not desirable with respect to addressing bone defects in relatively large sections and in force bearing sites [16]. In this study, we found that the stiffness of 3D-TCP scaffold was highly improved in comparison with conventionally made C-TCP scaffold, even though they are the same material with a similar pore size and porosity. It is reported that the mechanical properties of the commercial heterogeneous bone “Bio-Oss^®^” used in oral and maxillofacial clinic are 17.93 ± 0.292 Mpa, which are lower than our 3D-TCP scaffold [24]. The mechanical results in our study suggested that 3D regular interconnected porous structures facilitated the desirable mechanical properties. The PLGA component, which consists of polymer materials, is known to degrade faster than the β-TCP component [11]. Previous research has also reported that the stiffness of PLGA is too weak to be applied alone in hard tissue engineering, but it would be improved as the incorporated β-TCP content is increased concomitantly [15]. In order to enhance the mechanical property of PLGA and accelerate the degradation of β-TCP, we 3D printed a polymer composite of PLGA/TCP for use to repair minor bone defects in dental application [11,23]. From the viewpoint of attempting to increase the effective reinforcement, polymer organic fillers were also applied with β-TCP to assess improvements in relation to flexibility [25,26]. Interestingly, we found that 3D-PLGA/TCP composite scaffold was not broken during the compression tests (Figure 3a). We also found that the compressive modulus was significantly decreased compared to C-TCP and 3D-TCP groups (Figure 3b). This implied that the 3D printed PLGA/TCP was not able to withstand the likely pressure to be experienced at loading sites or in critical areas of bone defects. Nevertheless, 3DP flexible reinforcement could be applied for personalized small bone defect reconstruction in periodontology and implantology [27,28].

The biological response of stem cells on materials is affected by many factors, such as material composition, macropore size, micro surface morphology, and so on [29,30]. For dentists, hDPSCs is present as mesenchymal stem cells in dental pulp tissue, which are easier to obtain than other cells, and have excellent osteogenic differentiation properties [21]. When materials were applied directly, hDPSCs cells were embedded into the materials, which provides advantageous biomimetic microenvironments for cell adhesion, proliferation, and migration [22]. However, the adhesion and proliferation of cells normally occur only on the surface of conventional materials and cells cannot migrate deeply into materials due to the lack of well-interconnected pores [19,31]. Approaches facilitated by the use of live/dead staining have results indicating that C-TCP, 3D-PLGA/TCP, and 3D-TCP are biocompatible materials and could be used to provide stable environment for hDPSCs. hDPSCs viability at early time-points provides limited information related to cellular metabolic activity and should be used in conjunction with AlamarBlue. Thus, these developed 3D structures and materials could be used to strengthen cell migration and to facilitate their osteointegration, which has been identified as a critical factor involved in bone regeneration in vivo and, therefore, should be investigated in further research efforts.

The evaluation of the microenvironmental response from different scaffolds has significance with hDPSCs behavior in hard tissue regeneration. Microenvironments that are important in the dynamics underlying cell proliferation and osteogenic differentiation are affected by elemental ions released from the scaffolds [32,33]. Thus, in this study, we used AlamarBlue analysis to evaluate the effects of C-TCP, 3D-PLGA/TCP, and 3D-TCP scaffolds on cell proliferation. In vitro results indicated that hDPSCs in the 3D-TCP group proliferated markedly faster than cells in the C-TCP group after three days of co-culturing. Studies that examined the mechanisms of interaction between materials and organisms demonstrated that the behavior of cells in tissues and organs was not only regulated by gene sequences and proteins, but was also affected by external environmental factors [34,35,36]. Thus, the reasons that may help to explain the increase in cell metabolic activity may be related to fact that 3D printed β-TCP has a larger surface area than otherwise conventionally formed β-TCP. 3D printed β-TCP increases contact area with the culture medium, and makes the pores more suitable for cell metabolism and circulation. Moreover, we noted that the proliferation of hDPSCs in 3D-PLGA/TCP scaffold was significantly lower than in the other two groups on day 5 and 7. PLGA is a hydrophobic material and its degradation could create acidic microenvironments, resulting in reduced adhesion of hDPSC on 3D-PLGA/TCP scaffold [37]. Thus, its incorporation with β-TCP would be an approach that improves this performance [13,17]. However, cell metabolic activity and differentiation were affected to a certain extent from the application of β-TCP [15,17]. Therefore, this adverse observation can be hypothesized to have affected our research so that the PLGA element caused the lower cell proliferation in the 3D-PLGA/TCP group. A notable observation in Figure 5 was that the 3D-TCP group presented a higher ALP activity compared to the other two groups. In support of our findings in hDPSCs proliferation and ALP activity, studies have reported that the mechanism by which Ca^2+^ ions impact cell behavior is promoting cell proliferation and differentiation by activating chimeric Ag receptor T cells [32,38]. Calcium and phosphorous concentrations can also significantly affect the proliferation, differentiation, and mineralization of hDPSCs [39]. Researchers exploring extracellular Ca^2+^ ions, which occurred through the activation of Ca^2+^ influx, found that there was a promotion of osteogenic differentiation of hDPSCs [37,38]. As previously mentioned, the 3D printed scaffold had a larger surface area and stronger levels of permeation into the medium than conventionally manufactured scaffold. Therefore, we speculate that this may be the reason for the stronger osteogenic capacity of the C-TCP and 3D-TCP scaffolds. However, calcium and phosphate ions from β-tricalcium phosphate of the C-TCP, 3D-TCP, and 3D-PLGA/TCP scaffolds were not investigated and compared in this study. To fully elucidate and to prove that these 3D printed scaffolds could have enhanced hDPSCs proliferation and osteoinduction, further calcium and phosphate ions release from scaffold and RNA quantification of osteogenic markers should be investigated.

## 5. Conclusions

PLGA/TCP and β-TCP scaffold manufactured by 3DP have a potentially broad dental application for personalized levels of treatments. In our preliminary study and efforts herein, we found that both 3D-PLGA/TCP and 3D-TCP scaffolds demonstrated a regular and widely interconnected porous structure that facilitated the increased in cell proliferation, migration, and osteogenic differentiation. Thus, the introduction of 3D structure and β-TCP components not only contributed to better hDPSCs adhesion and proliferation, but also promoted osteogenic differentiation. Besides, compared to the C-TCP scaffold, we found that 3D-TCP scaffolds had higher mechanical properties which implied that the 3D network reinforces the compressive modulus. This is a crucial finding that can be used for customized reconstruction in critical bone defects. Moreover, this flexible feature of 3D-PLGA/TCP reinforcement could be applied for personalized bone augmentation in periodontology and implantology. Therefore, 3D-PLGA/TCP and 3D-TCP scaffolds incorporated with hDPSCs have a prospective potential in different clinical applications that can improve oral and maxillofacial bone regeneration.

## Figures and Tables

**Table 1:** The 3DP parameters for 3D-PLGA/TCP.

Description	Value
Layer height	375 µm
Pore diameter	300 µm
Nozzle diameter	400 µm
Printing speed	20 mm/s
Printing temperature	−30 °C

**Table 2:** The 3DP parameters for 3D-TCP.

Description	Value
Layer height	375 µm
Pore diameter	300 µm
Nozzle diameter	400 µm
Printing speed	5 mm/s
Printing temperature	15 °C

**Table 3:** Pore size and porosity.

Materials	Pore Size (μm)	Porosity (%)
C-TCP	375 ± 30.3	67.4 ± 2.7
3D-PLGA/TCP	362 ± 16.5	65.6 ± 5.0
3D-TCP	345 ± 9.1	72.5 ± 2.5

## Supplementary Materials

The following are available online at https://www.mdpi.com/1996-1944/13/14/3057/s1, Figure S1: Protein concentration of C-TCP, 3D-PLGA/TCP, and 3D-TCP scaffolds. (a) Standard curve of bovine serum albumin (BSA) protein. (b) BSA protein staining for calculating standard curve. (c) Quantified protein concentration of C-TCP, 3D-PLGA/TCP, and 3D-TCP scaffolds on day 3, 7, and 14.
