# Supplementary material for: In Vitro Mechanical and Biological Properties of 3D Printed Polymer Composite and β-Tricalcium Phosphate Scaffold on Human Dental Pulp Stem Cells
Source: Materials (Basel). 2020 Jul 8;13(14):3057. doi: 10.3390/ma13143057 (PMC7412522; doi:10.3390/ma13143057)
Supplement: Supplementary file 1 [file materials-13-03057-s001.pdf]

materials

MDPI

Article

In Vitro Mechanical and Biological Properties of 3D Printed Polymer Composite and β-Tricalcium Phosphate Scaffold on Human Dental Pulp Stem Cells

Shuaishuai Cao [1,2], Jonghyeuk Han [3], Neha Sharma [1,2], Bilal Msallem [1,2], Wonwoo Jeong [3], Jeonghyun Son [3], Christoph Kunz [1], Hyun-Wook Kang [3,*] and Florian M. Thieringer [1,2,*]

1 Department of Oral and Cranio-Maxillofacial Surgery, University Hospital Basel, Spitalstrasse 21, 4031 Basel, Switzerland; shuaishuai.cao@unibas.ch (S.C.); neha.sharma@usb.ch (N.S.); bilal.msallem@usb.ch (B.M.); christoph.kunz@usb.ch (C.K.)

2 Medical Additive Manufacturing Research Group, Department of Biomedical Engineering, University of Basel, Gewerbestrasse 16, 4123 Allschwil, Switzerland

3 Biomedical Engineering, School of Life Sciences, Ulsan National Institute of Science and Technology (UNIST), 50, UNIST-gil, Ulsan 44919, Korea; g14619@unist.ac.kr (J.H.); wwjeong@unist.ac.kr (W.J.); mgm4336@unist.ac.kr (J.S.)

* Correspondence: hkang@unist.ac.kr (H.-W.K.); florian.thieringer@usb.ch (F.M.T.)

Received: 18 May 2020; Accepted: 6 July 2020; Published: date

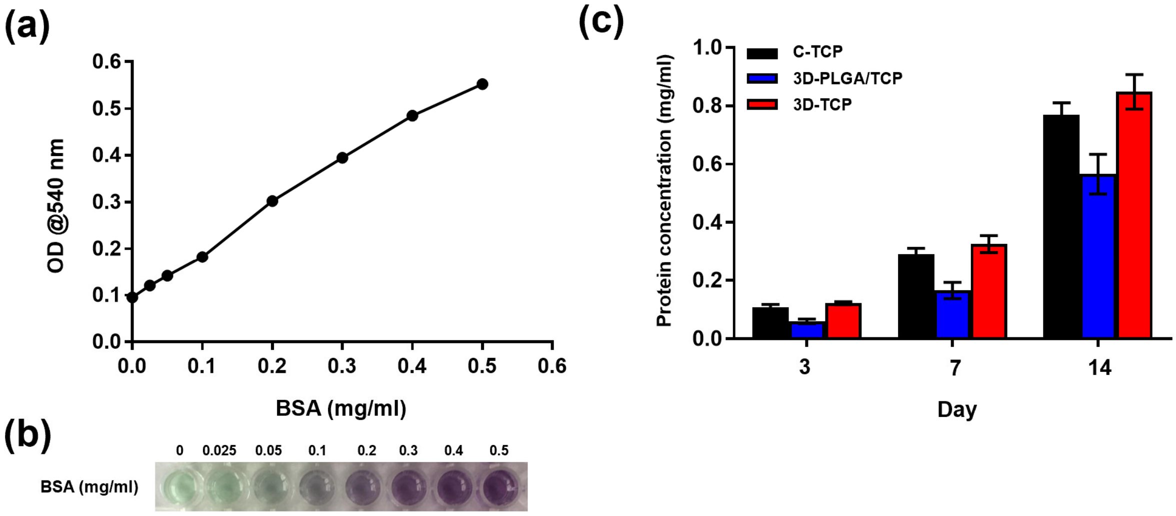

Figure S1. Protein concentration of C-TCP, 3D-PLGA/TCP, and 3D-TCP scaffolds. (**a**) Standard curve of bovine serum albumin (BSA) protein. (**b**) BSA protein staining for calculating standard curve. (**c**) Quantified protein concentration of C-TCP, 3D-PLGA/TCP, and 3D-TCP scaffolds on day3, 7, and 14.
